# Single-Locus and Multi-Locus Genome-Wide Association Studies Identify Genes Associated with Liver Cu Concentration in Merinoland Sheep

**DOI:** 10.3390/genes14051053

**Published:** 2023-05-08

**Authors:** Olusegun O. Adeniyi, Ivica Medugorac, Ewa Grochowska, Rolf-Alexander Düring, Gesine Lühken

**Affiliations:** 1 Institute of Animal Breeding and Genetics, Justus Liebig University Giessen, Ludwigstrasse 21, 35390 Giessen, Germany; olusegun.o.adeniyi@agrar.uni-giessen.de; 2 Population Genomics Group, Department of Veterinary Sciences, Ludwig Maximilian University Munich, Lena-Christ-Str. 48, 82152 Martinsried, Germany; i.medugorac@lmu.de; 3 Department of Animal Biotechnology and Genetics, Bydgoszcz University of Science and Technology, Mazowiecka 28 St., 85-084 Bydgoszcz, Poland; ewa.grochowska@pbs.edu.pl; 4 Institute of Soil Science and Soil Conservation, Interdisciplinary Research Center for Biosystems, Land Use and Nutrition (IFZ), Justus Liebig University Giessen, Heinrich-Buff-Ring 26-32, 35392 Giessen, Germany; rolf-alexander.duering@umwelt.uni-giessen.de

**Keywords:** liver copper concentration, SL-GWAS, ML-GWAS, candidate genes, Merinoland sheep

## Abstract

Economic losses due to copper intoxication or deficiency is a problem encountered by sheep farmers. The aim of this study was to investigate the ovine genome for genomic regions and candidate genes responsible for variability in liver copper concentration. Liver samples were collected from slaughtered lambs of the Merinoland breed from two farms, and used for measurement of copper concentration and genome-wide association study (GWAS). A total of 45,511 SNPs and 130 samples were finally used for analysis, in which single-locus and several multi-locus GWAS (SL-GWAS; ML-GWAS) methods were employed. Gene enrichment analysis was performed for identified candidate genes to detect gene ontology (GO) terms significantly associated with hepatic copper levels. The SL-GWAS and a minimum of two ML-GWAS identified two and thirteen significant SNPs, respectively. Within genomic regions surrounding identified SNPs, we observed nine promising candidate genes such as *DYNC1I2*, *VPS35*, *SLC38A9* and *CHMP1A*. GO terms such as lysosomal membrane, mitochondrial inner membrane and sodium:proton antiporter activity were significantly enriched. Genes involved in these identified GO terms mediate multivesicular body (MVB) fusion with lysosome for degradation and control mitochondrial membrane permeability. This reveals the polygenic status of this trait and candidate genes for further studies on breeding for copper tolerance in sheep.

## 1. Introduction

Sheep farming is continuously challenged with the risks of copper intoxication and deficiency. Copper is an essential trace element for many species. It serves as a cofactor for the proper functioning of some copper-containing enzymes, so-called ”cuproenzymes” such as copper-–zinc superoxide dismutase, cytochrome c oxidase and hephaestin which function in the anti-oxidation of free radicals, energy metabolism and iron metabolism, respectively [1,2]. However, sheep are known to be impaired in their ability to excrete excess copper from the liver, which leads to copper intoxication [3]. On the other hand, copper deficiency is also a known problem in sheep husbandry [4]. 

Hepatic copper levels in sheep have been classified into diagnostic levels of deficient, marginal, adequate, high, and toxic [5,6]. Deficient and high diagnostic levels of hepatic copper have been associated with poor performance and health status of sheep [7,8]. Liver copper levels have been reported to vary between as well as within sheep breeds [9,10]. A report on Suffolk and Texel sheep fed a similar diet showed that the coefficient of variation for liver copper concentration within each breed was 40% and 34%, respectively [9]. Furthermore, results of selection for low and high copper status in Scottish Blackface lambs have been reported by Woolliams et al. [11]. According to these authors, plasma copper concentration was higher in high copper lines than in low copper lines. In another report by Knowles et al. [12], variation in copper status was observed for two flocks of the Romney breed suggesting the availability of lines with distinct copper metabolism. These findings suggest that copper status within sheep breeds may be influenced by genetic factors. 

Association of genes such as copper transporter 1 (CTR1), chaperone for superoxide dismutase 1 (CCS), antioxidant 1 (ATOX1), metallothionein (MT), copper-transporting ATPase 1 (ATP7A) and copper-transporting ATPase 2 (ATP7B) with copper metabolism are well documented for humans [13,14,15,16,17]. CTR1, CCS and ATOX1 are proteins involved in copper transport from circulation, distribution to superoxide dismutase (SOD1) and transport to the secretory pathway and nucleus, respectively [14]. In addition, ATP7A/B supports the transfer of copper to the secretory pathways and mediates copper excretion by sequestration in vesicles/vacuoles, while MT functions as a copper scavenger in hepatocytes [14,15,17]. Variations in liver copper levels have been reported for humans with mutations in the ATP7A and/or ATP7B genes that result in Menkes and Wilson disease, respectively [18,19]. Likewise, mutation in the copper metabolism MURR1 domain-containing 1 (COMMD1) gene has been implicated in copper toxicity in dogs [2]. However, information on candidate genes and genetic markers associated with within-breed variation in liver copper concentration of sheep is unknown.

The Illumina Ovine 50K SNP chip is a powerful tool to analyze the sheep genome with respect to an association with diverse traits as confirmed in various single-locus genome-wide association studies (SL-GWAS) [20,21,22,23] and multi-locus GWAS (ML-GWAS) [24,25] in animals. SL-GWAS aim to identify associations of single nucleotide polymorphisms (SNPs) with varying phenotypes of individuals by assessing the differences in allelic frequencies of their genetic variants [26,27]. This is currently implemented in various mixed linear model methods such as MLMA [28], EMMAX [29] and GEMMA [30]. These methods are usually subjected to Bonferroni correction due to multiple testing, which may result in the exclusion of some important loci with small effects. In this context, ML-GWAS is a better alternative because it does not require the conservative Bonferroni correction, therefore leading to the identification of more trait-associated markers especially for polygenic traits [25,31]. Moreover, an earlier report indicated that a multi-locus GWAS approach is better suited for the analysis of complex traits [32]. As a solution, several ML-GWAS models have been developed, such as multi-locus random-SNP-effect mixed linear model (mrMLM) [33], the fast multi-locus random-SNP-effect mixed linear model (FASTmrMLM) [34], the fast multi-locus random-SNP-effect efficient mixed-model association (FASTmrEMMA) [35], polygenic-background-control-based least angle regression plus empirical Bayes (pLARmEB) [36], polygenic-background-control-based Kruskal–Wallis test plus empirical Bayes (pKWmEB) [37], and Iterative sure independence screening expectation maximization Bayesian least absolute shrinkage and selection operator (ISIS EM-BLASSO) [38]. These methods are implemented in two phases. The first phase involves the selection of potentially associated markers after analyses using various algorithms. For the second phase, all the effects in a model harbouring the selected markers are estimated by empirical Bayes, after which further identification by a likelihood ratio test for true quantitative trait nucleotides (QTNs) of all the non-zero effects is performed. 

To the best of our knowledge, a genome-wide analysis regarding liver copper concentration in sheep has not been conducted until now. Therefore, the aim of this study was to identify candidate genes associated with variability in hepatic copper concentration in sheep using both SL-GWAS and ML-GWAS methods. This analysis was performed using liver samples from slaughtered lambs of the Merinoland sheep breed, a breed of high economic importance in Germany.

## 2. Materials and Methods

### 2.1. Sample and Data Collection

The animals were kept under standard farming regulations on two private sheep farms in Bavaria. For this study, only male lambs were sampled after slaughter for human consumption. Documented information on the date of birth and date of slaughter was obtained from the farmers. Regarding farm 1, 89 lambs from the flock with ages between 4 and 5.5 months were sampled after slaughter in batches over a period of 46 days. They were kept indoors with unlimited access to their dams and fed ad libitum with grass silage and compound feed consisting of lucerne pellets (5.53%), concentrate pellets (58.84%), barley (22.93%) and beet pulp (12.70%). Concentrate feeding of lambs started 14 days after birth. All dams had unhindered access to grass silage with occasional input of the above-mentioned compound feed. A total of 45 lambs were sampled from the flock located at farm 2. These lambs were kept on pasture and under uniform feeding conditions until slaughter. In addition, fresh water was made available ad libitum for all animals by the farmers. The nutrient composition of the feed, and the mineral content of the grass silage, components of compound feed, and pasture grass were determined by a nutrient analysis laboratory (Intertek Food Services GmbH, Linden, Germany) (Table S1a,b). Liver samples from all lambs were collected directly after slaughter by cutting approximately 20 g of sheep liver from the tip of the *Lobus caudatus*, packed separately in plastic tubes, labelled and stored at −20 °C prior to the determination of liver copper concentration and DNA extraction.

### 2.2. Determination of Liver Copper Concentration

Liver samples were freeze-dried, pulverized and stored in airtight tubes. Duplicate samples of approximately 0.5 g were digested in 5 mL deionized H_2_O, 5 mL HNO_3_ and 3 mL H_2_O_2_ with a StarT-1500 microwave oven (MLS GmbH Cooperation, Leutkirch, Germany). Conditions for the microwave digestion system are shown in Table S2. Digested samples were filtered into a 50 mL flask and filled up to a volume of 50 mL with deionized water. The copper concentration was determined with an inductively coupled plasma-optical emission spectrometer (ICP-OES; Agilent 720ES, Darmstadt, Germany) at a wavelength of 327.4 nm. Used operating parameters for the ICP-OES were published elsewhere [39]. For quality assurance reagent blanks were measured and a detection limit corresponding to 0.039 mg/kg was calculated (limit of detection = mean blank + 3 standard deviations of the mean blank value). Furthermore, certified reference materials (Bovine Liver ERM-BB185, European Commission, Joint Research Centre, Geel, Belgium) were used for testing the precision of measurement. The mean recovery percentage was 91.4% ± 2.6.

### 2.3. Genotyping and Quality Control

DNA was extracted from liver samples using the Macherey-Nagel NucleoSpin Tissue Kit (Düren, Germany) according to the manufacturer’s instructions. All 134 liver samples were genotyped with the Illumina Ovine 50k SNP BeadChip. Quality control (QC) was performed to exclude loci with minor allele frequency (<5%), SNPs with a low call rate (<95%), samples with more than 5% of missing genotypes, and SNPs for which Hardy–Weinberg equilibrium test *p*-values were lower than 1.0 × 10^−6^. After QC a total of 45,511 SNPs and 130 samples were left for analysis.

### 2.4. Single-Locus and Multi-Locus Genome-Wide Association Analyses

SL-GWAS and ML-GWAS were performed for all samples and data obtained after QC. All 130 samples were used to estimate the genome-wide association of markers with liver copper concentration. To perform a single-locus GWAS, genetic relatedness was estimated using “pcrelate” and “pcrelateToMatrix” functions in the GENESIS R package [40]. Next, a linear mixed model analysis for quantitative phenotypes was implemented using the same software. A genetic relationship matrix was included in the analysis as a random effect to account for cryptic relatedness. The model includes: $y=X\beta +Z_{1}a+Z_{2}c+e$ where y is the vector of observed liver copper concentration (mg/kg dry matter, DM) at slaughter, ß is a vector of fixed effects including the intercept term, farm, age at slaughter, a is the vector of the additive effect (fixed) of the candidate SNP to be tested for association, c is the vector of polygenic effect (random) of all markers (as captured by the genetic relationship matrix calculated using all SNPs), and e is a vector of residuals. X, Z_1_ and Z_2_ are the incidence matrices relating observations to β, a and c, respectively. Due to the conservative nature of Bonferroni threshold [41] and possibility that associated SNP effects can be missed [42], a false discovery rate (FDR) *p*-value threshold was used [43]. FDR was set at 0.01, with the *p*-value level of significance defined as *p* = N/M × 0.01, where N is the number of SNP markers with *p*-values below the FDR threshold and M represents the total number of SNPs [44]. A Manhattan plot of -log_10_(*p*-value) was constructed with the package “ggplot” in R [45]. SNPs above the given *p*-value threshold were considered significant and selected for further analysis. The proportion of total variability explained by SNPs (V_G_) and error variance (V_E_) were computed using “varCompCI” function in GENESIS R package [40]. Estimation of heritability was performed from the variance components with the formula: h^2^ = V_G_ / (V_G_ + V_E_) according to Yang et al. [46].

Regarding ML-GWAS analysis, the mrMLM v4.0.2 [47] software implemented in R was used to determine SNPs associated with liver copper concentration in Merinoland sheep. Five ML-GWAS methods including mrMLM, FASTmrMLM, FASTmrEMMA, pLARmEB, and ISIS EM-BLASSO were performed according to methods described by Wang et al. [33], Tamba and Zhang [34], Wen et al. [35], Zhang et al. [36], and Tamba et al. [38], respectively. In the first stage and for the selection of potential SNPs, all SNPs were treated as random effects in the five methods. In the second stage, all selected SNPs in the first stage of each method were placed into one multi-locus model and the markers with effects above the logarithm of odds (LOD) threshold value were regarded as possible candidate SNPs associated with liver copper concentration. For all methods except pLARmEB, the critical *p*-values for the selection of SNP in the first stage were set at default. These values were employed in order to maximize power for SNP detection as suggested by the respective authors [33,34,35,38]. The default values were 0.01 for mrMLM, 0.01 for FASTmrMLM, 0.005 for FASTmrEMMA, and 0.01 for ISIS EM-BLASSO. With regards to pLARmEB analysis, a value of 50 was set as the number of potentially associated markers selected by least angle regression (LARS) according to Zhang et al. [36]. The critical LOD score was set to 3 in the second stage according to previous reports using ML-GWAS methods [24,25]. Covariates of farm and age at slaughter were included in the analysis, which was corrected for cryptic relatedness using K (genomic relatedness) matrix calculated using the R package mrMLM v4.0.2. To increase the accuracy of this result, SNPs identified by at least two ML-GWAS methods were selected for further analysis.

### 2.5. Gene Annotation and Enrichment

Genes located within 0.5 megabase (Mb) regions upstream and 0.5 Mb downstream of putative SNPs after SL-GWAS and ML-GWAS were identified according to the UCSC genome browser [48] with the selection of the Oar_v4.0 assembly in the National Center for Biotechnology Information (NCBI). Functional enrichment analysis was performed on all genes identified by both GWAS methods using the Database for Annotation, Visualization and Integrated Discovery (DAVID) software (https://david.ncifcrf.gov; accessed on 23 February 2023) [49,50,51]. The human gene annotation was selected for analysis due to a higher number of recognized genes in comparison to sheep gene annotations. The gene ontology (GO) annotation category including biological process (BP), cellular component (CC) and molecular function (MF) terms, with the parameter set to a minimum of two genes, was investigated in this study. Using the Fishers exact test, GO TERMS of enriched genes with a *p*-value threshold of ≤0.05 were considered significant [52]. A subset of genes with functional relationship to cellular transport and excretion using available gene annotations from GeneCards (http://www.genecards.org (accessed on 23 February 2023)) and Uniprot (http://www.uniprot.org (accessed on 23 February 2023)) databases were considered as functional candidate genes. Additionally, a second subset of all genes was considered as positional candidate genes when harbouring a significant SNP or located near such an SNP.

## 3. Results

### 3.1. Liver Copper Levels and Estimated Heritability

Descriptive statistics of hepatic copper concentration and age of slaughtered lambs whose samples were included in the SL-GWAS and ML-GWAS analyses are shown in Table 1. A heritability of 0.67 ± 0.29 was estimated for this trait.

### 3.2. Identification of Genomic Regions and Candidate Genes 

**Figure 1:**
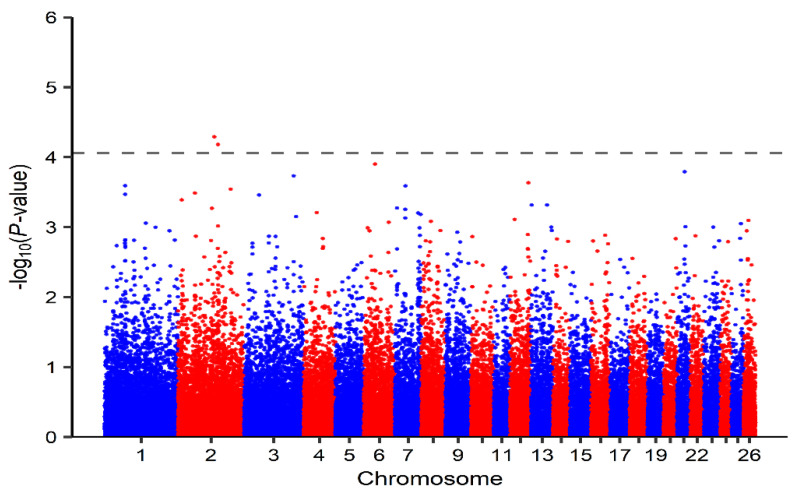
Manhattan plot of single-locus genome-wide association study (SL-GWAS) of liver Cu concentration for Merinoland sheep. The dashed line represents the FDR threshold (−log_10_(*p*-value) = 4.06).

**Figure 2:**
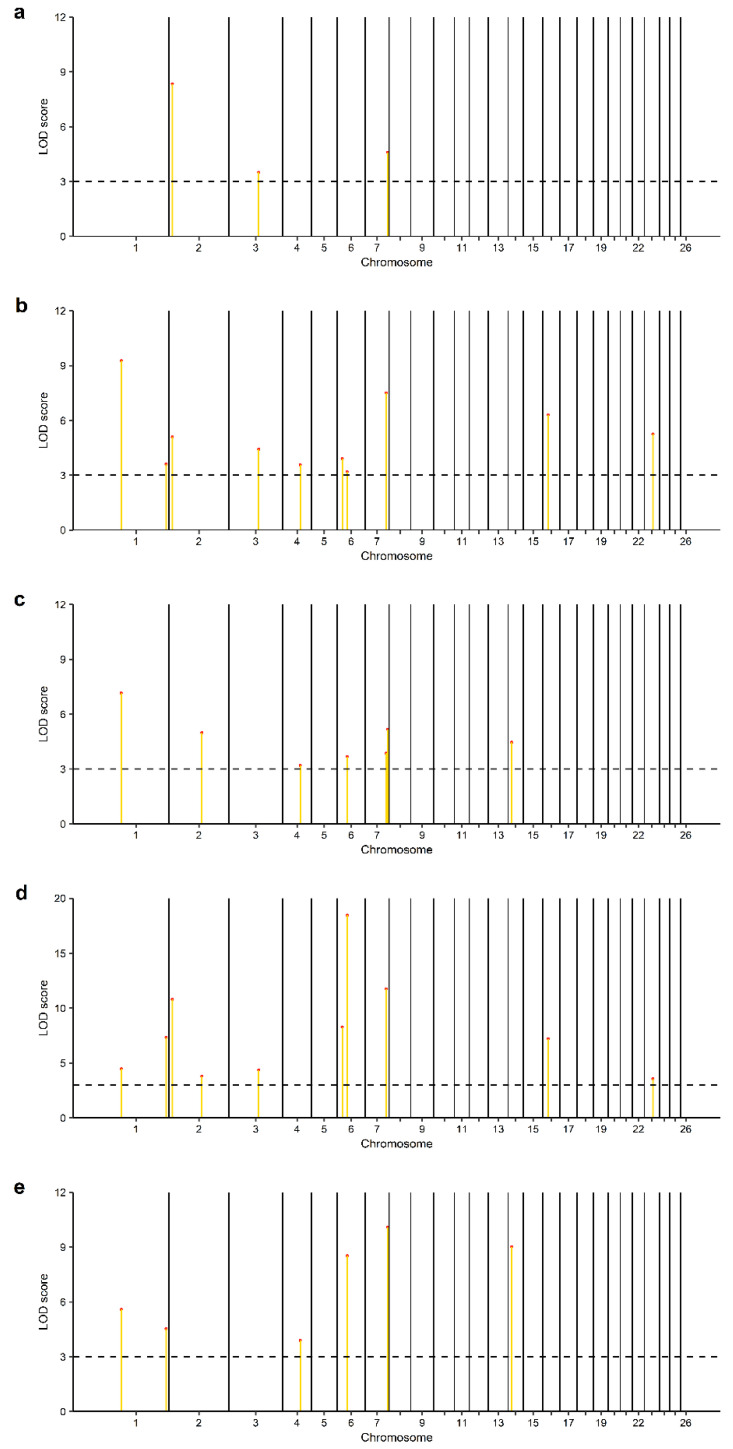
Manhattan plots of the five multi-locus genome-wide association studies (ML-GWAS) (**a**) mrMLM, (**b**) FASTmrMLM, (**c**) FASTmrEMMA and (**d**) pLARmEB, (**e**) ISIS EM-BLASSO for liver Cu concentration in Merinoland sheep. Yellow lines with red dots represent significant SNPs above the LOD thresholds. Dashed lines represent the thresholds for significance (LOD > 3).

Relating to the SL-GWAS analysis of markers associated with liver copper concentration in this study, two significant SNPs (OAR2_145591151.1 and s62875.1) located on chromosome 2 and below the given threshold (*p*-value = 8.77 × 10^−5^; −log_10_(*p*-value) = 4.06; λ = 0.98) were observed (Table 2; Figure 1). Likewise, 35 significant SNPs above the LOD score cutoff value of 3 were observed after the performance of ML-GWAS methods (Table S3). Among these, 13 significant SNPs distributed on chromosomes 1, 2, 3, 4, 6, 7, 14, 16 and 23 (Table 3; Figure 2a–e) were detected by a minimum of two ML-GWAS methods. The two SNPs observed using the SL-GWAS were also identified by ML-GWAS. Of these, only one SNP (OAR2_145591151.1) was observed by more than one ML-GWAS method. Concerning candidate genes identified with SL-GWAS, 4 positional candidate genes (Table 2) within putative SNP regions were detected. Furthermore, two of these candidate genes were identified as both positional and functional. As regards ML-GWAS and within possible SNP regions associated with liver copper concentration, we observed 21 potential candidate genes (Table 3). Among them were 9 functional candidate genes, with 4 genes identified as both positional and functional candidate genes. 

Finally, our results revealed a total of 16 GO terms, for example integral component of membrane, lysosomal membrane, hydrogen ion transmembrane transport, mitochondrial inner membrane and sodium:proton antiporter activity were significantly enriched (*p* ≤ 0.05; Table 4).

## 4. Discussion

Copper homoeostasis is less efficient in sheep compared to other farm animals such as cattle and goats [3]. This impairment can more easily lead to copper intoxication or deficiency and is reported to vary within and between breeds [1,9,10]. The study of copper-related genes is well documented [14,15,16,17]. However, there is a scarcity of information on the gene or genes responsible for the control of hepatic copper levels in sheep. This study aimed at identifying possible SNP markers and candidate genes associated with liver copper variation in Merinoland sheep. Measured hepatic copper concentrations were within the marginal and normal levels expected for sheep [6]. The estimated, high heritability (h^2^ ± SE = 0.67 ± 0.29) confirmed that this trait is largely influenced by genetic factors. This value is in the same range as estimated in Merino sheep by Judson et al. [53] (h^2^ ± SE = 0.60 ± 0.32; *n* = 208). However, the high SE for h^2^ observed in our study indicates that the heritability value may be inflated due to cryptic relatedness since SNP-based heritability is reported to increase with a higher number of related samples [46]. Therefore, further work with a larger sample size of unrelated animals is needed to validate this finding.

Among the candidate genes identified in this study, three genes including dynein cytoplasmic 1 intermediate chain 2 (*DYNC1I2*), retromer complex component (*VPS35*) and solute carrier family 38 member 9 (*SLC38A9*) located on chromosomes 2, 14 and 16 have been reported to indirectly influence cellular transport and excretion [54,55,56]. The *DYNC1I2* gene expresses a subunit of the cytoplasmic dynein motor protein which is important for microtubule-based transport towards the minus end [57]. Cytoplasmic dynein transports cargoes such as cytoskeleton filaments, endosomes and lysosomes and has been reported to bind dynactin, an important protein complex associated with dynein activity through its p150Glued subunit [58,59]. Likewise, dynactin is reported to interact with the copper P-type ATPase (ATP7B) via its subunit p62 [60]. Interestingly, lysosomal copper exocytosis has been directly associated with dynactin in a report by Polishchuk et al. [61]. Findings in this report suggest that the targeting of ATP7B to the canalicular surface in hepatocyte cells, which precedes lysosomal exocytosis, requires copper-dependent interaction with the p62 dynactin subunit. This suggests that changes in the activity of cytoplasmic dynein motor protein may determine hepatic copper levels by impacting lysosomal exocytosis. Besides VPS29 and VPS26, VPS35 is one of three core proteins of the retromer complex, involved in cargo sorting and endosomal trafficking including the homeostasis of transmembrane proteins located at the plasma membrane and within endosomes/ lysosomes [62]. The retromer complex is reported to bind sorting nexin 3 (SNX3) resulting in the cargo recognition and binding of divalent metal transporter 1 (also known as solute carrier family 11 member 2) [62,63]. DMT1 has been implicated in copper ion transport in the intestine with a reduced expression resulting in lower copper absorption [64]. In addition, the VPS35 associates with FAM21 (a subunit of the WASH complex) and aids the recruitment of the WASH complex to endosomes [65,66,67]. Earlier findings suggest that copper metabolism MURR1 domain–containing 1 (COMMD1) protein, which functions as a copper chaperone and binds to the copper-transporting P-type ATPases ATP7A/ATP7B [68,69], interacts with the WASH complex and modulates copper-dependent trafficking of ATP7A [68]. For perspective, ATP7A is an important copper transporter that regulates the absorption of copper in the intestinal tract [70]. Yeast cells are considered an ideal model for the genetic analysis of mammalian genomes [71,72]. Interestingly, a report by Sowada et al. [73] showed increased copper sensitivity of yeast cells lacking VPS35. Therefore, the interaction of VPS35 with the DMT1 and WASH complex may have an influence on liver copper concentration in Merinoland sheep. The *SLC38A9* gene encodes a lysosomal amino acid transporter protein reported in the mechanistic target of rapamycin complex 1 (mTORC1) signalling [56,74,75]. The mTORC1 controls transcription factor EB (TFEB) which regulates the expression of genes involved in lysosomal degradation and biogenesis such as vacuolar H^+^-ATPase (V-ATPase) and metallothionein 1 (MT1) [76,77]. The MT1 protein has been identified as a chaperon involved in copper homoeostasis [78]. This indicates that the involvement of SLC38A9 in mTORC1 signalling and TFEB expression may impact liver copper levels in Merinoland sheep. Notably, TFEB expression has been associated with lysosomal copper exocytosis [61]. In this report, increased TFEB expression in hepatocyte cells resulted in increased lysosomal copper exocytosis suggesting a possible influence of TFEB expression on liver copper exocytosis.

Regarding GO analysis, our findings revealed that lysosomal membrane and midbody were enriched GO terms in this study. Genes observed in these GO terms including *VPS35, SLC38A9* and charged multivesicular body protein 1 A (*CHMP1A*) are reportedly involved in cellular transport and excretion [62,75,79]. Generally, the endosomal sorting complex required for transport (ESCRT) is important for the sorting and delivery of ubiquitinated cargo such as membrane transporters to the lysosome for degradation [80]. The CHMP1A is the member of the ESCRT-III family required for the formation of multivesicular bodies (MVBs), which fuse with lysosomes for the degradation of its content [79,81]. Additionally, the ESCRT-III is involved in the abscission of budding vesicles which mature into MVBs [80]. These findings suggest a possible influence of ESCRT on the degradation of copper transporters and consequently liver copper concentration. Furthermore, some genes including matrix AAA peptidase subunit, paraplegin (*SPG7*) and ATP synthase membrane subunit f (*ATP5MF*) were observed as functional genes involved in the mitochondrial inner membrane, which is a significant GO term observed in this study. The *SPG7* and *ATP5MF* genes are implicated in the opening of the mitochondrial permeability transition pore (PTP) [82,83,84]. The opening of the PTP results in loss of ATP production, mitochondrial swelling and cell death due to cytosolic calcium overload and oxidative stress [83]. Interestingly, another gene (solute carrier family 25 member 12 (*SLC25A12*)) identified in this GO term is also required for the calcium-dependent maintenance of ATP homoeostasis in the mitochondria [85,86]. The mitochondria are important cellular organelles implicated in copper homeostasis [87]. Earlier reports suggest that copper toxicity in cells results in swelling and rupture of the mitochondria, as well as induction of oxidation stress [3,88]. According to Bernardi et al. [89], mitochondrial swelling is associated with changes in the inner mitochondrial membrane permeability and consequent expansion of the inner membrane matrix. In the report by Haywood et al. [3], mitochondria swelling in hepatocytes from the North Ronaldsay sheep breed, which is considered highly susceptible to copper intoxication, was observed at a lower liver copper concentration when compared to a less susceptible sheep breed (Cambridge sheep). These observations suggest a possible association of hepatic copper levels with gene functions regulating mitochondrial membrane permeability. Finally, the GO terms hydrogen ion transmembrane transport and sodium:proton antiporter activity were also strongly enriched with involved genes such as solute carrier family 9 member B1 (*SLC9B1*) and solute carrier family 9 member B2 (*SLC9B2*). These genes encode Na+/H+ exchangers (NHEs) important in cytoplasmic and organelle pH [90]. The *SLC9B1* gene is predominantly expressed in the testis while *SLC9B2* is highly expressed in the liver and brain. In earlier studies on yeast NHE (Nhx1p), it was observed that Nhx1p localized to the trans-Golgi network compartments, late endosomes, and recycling endosomes, is critical for protein sorting and endocytic pathway [91,92]. In another report, Nhx1 was found to aid MVB fusion with lysosomes [93]. These findings suggest that NHEs may influence endosomal sorting and trafficking of important copper transporters and consequently copper concentration in hepatocytes.

## 5. Conclusions

The results revealed a number of identified SNPs potentially contributing to variability in hepatic copper levels in Merinoland sheep. The identified regions surrounding these SNPs harbour some promising functional candidate genes such as *DYNC1I2*, *VPS35*, *SLC38A9* and *CHMP1A* associated with endosomal cargo sorting and trafficking, as well as lysosomal transport. Additionally, genes such as *SPG7, ATP5MF* and *SLC25A12* were identified as functional genes involved in mitochondrial membrane permeability which has been associated with copper toxicity. Likewise, the genes *SLC9B1* and *SLC9B2* which are involved in luminal and intraluminal pH and MVB fusion to lysosome, were observed as potential candidate genes influencing hepatic copper levels in Merinoland sheep. In total, nine promising candidate genes were observed in this study. These genes need to be further investigated to ascertain their involvement in liver copper variation in Merinoland sheep in particular, and other sheep breeds in general. This study provides evidence for a polygenic trait with high heritability and delivers promising clues for further studies to identify potential causal variants that may be associated with variation in copper tolerance in sheep and used for practical breeding.

## Figures and Tables

**Table 1:** Descriptive statistics and estimated heritability of liver copper concentration for Merinoland sheep lambs used in SL-GWAs and ML-GWAS analyses.

Farm	N ^1^	Mean (SE) Cu (mg/kg DM)	Min	Max	Mean (SE) Age at Slaughter (Days)	Min	Max	h^2^(SE) ^2^
1	89	159.54 ± 5.22	67.67	273.42	150.76 ± 1.30	119	165	0.67(0.29)
2	41	79.05 ± 6.80	20.57	188.28	136.78 ± 3.04	89	172	

^1^ N refers to number of samples; ^2^ h^2^ refers to heritability of liver copper concentration.

**Table 2:** Significant SNPs and genes associated with liver Cu concentration for Merinoland sheep using SL-GWAS.

CHR	SNP ^1^	Position	*p*-Value	Genes ^2^ (1 Mb)
2	OAR2_145591151.1	136897136	5.11 × 10^−5^	***SLC25A12*, *DYNC1I2***
2	s62875.1	150905130	6.59 × 10^−5^	*ACVR1C*, *ACVR1*

^1^ SNP positions are based on Oar_v4.0 assembly in NCBI. ^2^ Candidate genes: underlined = positional candidate genes; bold = functional candidate genes within of SNP within a 1 Mb region.

**Table 3:** Significant SNPs and genes associated with liver Cu concentration for Merinoland sheep using ML-GWAS.

CHR	SNP	Position (BP) ^1^	Genes (1 Mb) ^2^	ML-GWAS Methods ^3^
1	s66850.1	77025732	*GPR88*, ***ATP5MF***	2, 3, 4, 5
1	OAR1_285395930.1	263664109	*COL6A1, COL6A2*	2, 4, 5
2	s05644.1	14172465	*FRRS1L*	1, 2, 4
2	OAR2_145591151.1 *	136897136	***SLC25A12, DYNC1I2***	3, 4
3	OAR3_132833292.1	124516955	*KITLG*	1, 2, 4
4	OAR4_77358490.1	73042599	*ZNF804B*	2, 3, 5
6	s42668.1	21789865	*CENPE, **SLC9B2, SLC9B2***	2, 4
6	OAR6_47263223.1	42315913	*GBA3*	2, 3, 4, 5
7	s25674.1	87187746	*NRXN3*	2, 3, 4
7	OAR7_101357352.1	93147667	-	1, 3, 5
14	OAR14_14650208.1	14404415	***SPG7, CHMP1A*** *, SHCBP1, **VPS35***	3, 5
16	OAR16_25377664_X.1	23278766	*IL6ST, **SLC38A9***	2, 4
23	OAR23_37101686.1	35080540	*GREB1L*	2, 4

^1^ SNP positions are based on Oar_v4.0 assembly in NCBI. ^2^ Candidate genes: underlined = positional candidate genes; bold = functional candidate genes of SNP within a 1 Mb region. ^3^ ML-GWAS Methods: 1 = mrMLM, 2 = FASTmrMLM, 3 = FASTmrEMMA, 4 = pLARmEB, 5 = ISIS EM-BLASSO; * SNP identified by both SL-GWAS and ML-GWAS.

**Table 4:** Enriched GO terms determined by DAVID from genes identified in regions (1 Mb) of SNPs for liver copper variation in Merinoland sheep using SL-GWAS and ML-GWAS.

Category	Term	Genes	*p*-Value ^1^
GOTERM_MF_DIRECT	GO:0016361~activin receptor activity, type I	*ACVR1, ACVR1C*	0.006
GOTERM_CC_DIRECT	GO:0005765~lysosomal membrane	*COL6A1, CHMP1A, VPS35, SLC38A9*	0.007
GOTERM_CC_DIRECT	GO:0048179~activin receptor complex	*ACVR1, ACVR1C*	0.008
GOTERM_BP_DIRECT	GO:1902600~hydrogen ion transmembrane transport	*SLC9B1, ATP5MF, SLC9B2*	0.010
GOTERM_CC_DIRECT	GO:0005743~mitochondrial inner membrane	*SPG7, SLC25A12, ATP5MF, SLC9B2*	0.014
GOTERM_CC_DIRECT	GO:0016021~integral component of membrane	*ACVR1, KITLG, FRRS1L, GREB1L, NRXN3, SLC38A9, SPG7, IL6ST, SLC25A12, SLC9B1, ATP5MF, SLC9B2*	0.014
GOTERM_MF_DIRECT	GO:0015385~sodium:proton antiporter activity	*SLC9B1, SLC9B2*	0.015
GOTERM_CC_DIRECT	GO:0030496~midbody	*CENPE, CHMP1A, SHCBP1*	0.016
GOTERM_CC_DIRECT	GO:0005828~kinetochore microtubule	*CENPE, CHMP1A*	0.021
GOTERM_BP_DIRECT	GO:0032924~activin receptor signalling pathway	*ACVR1, ACVR1C*	0.023
GOTERM_CC_DIRECT	GO:0043235~receptor complex	*ACVR1, ACVR1C, IL6ST*	0.023
GOTERM_CC_DIRECT	GO:0097228~sperm principal piece	*SLC9B1, SLC9B2*	0.035
GOTERM_MF_DIRECT	GO:0019838~growth factor binding	*ACVR1C, IL6ST*	0.040
GOTERM_MF_DIRECT	GO:0030020~extracellular matrix structural constituent conferring tensile strength	*COL6A2, COL6A1*	0.045
GOTERM_BP_DIRECT	GO:0007080~mitotic metaphase plate congression	*CENPE, CHMP1A*	0.047
GOTERM_BP_DIRECT	GO:0001755~neural crest cell migration	*ACVR1, KITLG*	0.049

^1^ *p* = *p*-value significant at 0.05; Please note that GOTERM_MF, GOTERM_CC and GOTERM_BP refer to molecular function, cellular component and biological process, respectively.

## Data Availability

Raw data are available on request from the authors.

## Supplementary Materials

The following supporting information can be downloaded at: https://www.mdpi.com/article/10.3390/genes14051053/s1, Table S1a: Nutrient composition of compound feed fed to Merinoland sheep on farm 1; Table S1b: Mineral content of grass, compound feed and pasture grass fed to Merinoland sheep on farm 1 and 2; Table S2: Operating conditions for microwave-assisted extraction of freeze-dried liver samples; Table S3: Significant SNPs associated with liver Cu concentration in Merinoland sheep detected by ML-GWAS analysis methods (LOD score > 3).
